# Men’s Facial Width-to-Height Ratio Predicts Aggression: A Meta-Analysis

**DOI:** 10.1371/journal.pone.0122637

**Published:** 2015-04-07

**Authors:** Michael P. Haselhuhn, Margaret E. Ormiston, Elaine M. Wong

**Affiliations:** 1 School of Business Administration, University of California Riverside, Riverside, California, United States of America; 2 Organisational Behaviour Area, London Business School, London, United Kingdom; University of Goettingen, GERMANY

## Abstract

Recent research has identified men’s facial width-to-height ratio (fWHR) as a reliable predictor of aggressive tendencies and behavior. Other research, however, has failed to replicate the fWHR-aggression relationship and has questioned whether previous findings are robust. In the current paper, we synthesize existing work by conducting a meta-analysis to estimate whether and how fWHR predicts aggression. Our results indicate a small, but significant, positive relationship between men’s fWHR and aggression.

## Introduction

A rapidly growing body of research published in journals ranging from biology (e.g., *Biology Letters*, *Proceedings of the Royal Society*) to psychology (e.g., *Psychological Science*) has established links between men’s facial width-to-height ratio (bizygomatic width divided by upper facial height; *fWHR*) and a wide range of behaviors. For instance, researchers have demonstrated that greater fWHRs are associated with socially undesirable behaviors in men, including being less trustworthy, less cooperative in the context of intra-group competition, and more prejudiced [1–3]. Research has also identified positive correlates of fWHR—men with greater fWHRs are more cooperative in the context of inter-group competition, are better negotiators in competitive bargaining, and firms led by CEOs with greater fWHRs achieve superior financial performance [3–5].

The theoretical basis for much of this research stems from Carré and McCormick’s [6] seminal paper in which they observe a positive link between fWHR and aggression in men. Specifically, men with greater facial width-to-height ratios were more likely to react aggressively to a perceived slight by others, and hockey players with greater facial ratios were more likely to be penalized in hockey games than were men with smaller facial ratios. Similarly, men’s facial ratios predict other forms of social aggression including self-interested behavior and the tendency to violate trust in an economic game [2]. Interestingly, it does not appear that fWHR is predictive of any changes in aggressive behavior, in particular, and behavioral or psychological outcomes more generally, in women (e.g., [2, 6, 7]; see [8] for an exception). This exclusive relationship in men is important from an evolutionary perspective as it suggests that men’s fWHR is an honest signal of superiority in intra-sexual conflict.

Recent research has sought to explain the underlying mechanisms by which men’s fWHR relates to aggressive behavior. One perspective is that men’s fWHRs serve as proxies for other psychological or biological characteristics that lead men with various facial structures to act differently. For instance, researchers have theorized that testosterone exposure at puberty may underlie intra-sex differences in fWHR [6], and this increased testosterone exposure may, in turn, lead to more aggressive behavior among men with greater fWHRs (cf. [9]). Other research has provided empirical evidence of a positive relationship between men’s fWHR and their baseline levels of testosterone [10].

A second perspective on the relationship between fWHR and aggressive behavior is that men are treated differently by others as a result of their facial characteristics [11]. Indeed, researchers have found that men with greater facial ratios are perceived to be more aggressive and less trustworthy [2, 12–13]. Consequently, these observers’ perceptions of men with greater fWHRs can elicit behaviors from these men that are consistent with the observers’ initial expectations. If, for example, people generally defer to men with greater facial ratios in order to avoid an aggressive confrontation, these men may “learn” to feel more powerful and less deferential over time as a result [7], see also [14–16]. According to this view, men’s facial structure may not have an inherent relationship to their aggressive behavior, but rather serves as a social cue that shapes their interactions with others over time.

Regardless of the source of the link between fWHR and aggressive tendencies, numerous researchers have explicitly built directly on this previous work, finding, for example, that men with greater fWHRs are better fighters, more likely to deceive others, and are less likely to die from contact violence [6–7, 17–18].However, despite the powerful influence of work relating fWHR and aggression, several articles have failed to find a statistically significant relationship between the two constructs (e.g., [19–20]). This has led some to question whether previous findings are merely due to Type 1 error [20] and, more broadly, has called the theoretical foundation of previous fWHR research into question. Further, studies examining the fWHR-aggression link have employed varied measures (e.g., scale and behavioral measures), samples from various nations, and varied sample sizes, leading to difficulties in estimating the magnitude of any possible relationship. In the current paper, we address the questions of whether and how fWHR relates to aggression by conducting a meta-analysis of existing research. The results of our analysis suggest that men’s fWHR is a small, but significant, predictor of aggression.

## Materials and Methods

### Identification of studies

A registered review protocol does not exist for this systematic review. We identified studies for our meta-analysis in three steps. First, we collected all papers involving research on fWHR. Specifically, we searched the PsycINFO database for the terms “facial-width-to-height ratio” and “fWHR.” Additionally, we individually searched journals that have published work on fWHR but are not indexed in the PsycINFO database (e.g., *PLoS One; Evolution and Human Behavior; Biology Letters; Proceedings of the Royal Society B*: *Biological Sciences*). We also used Google Scholar to locate published and unpublished manuscripts that cite seminal work on fWHR and aggression (i.e., [6]). Moreover, we contacted corresponding authors of many of these papers (N = 41) to request copies of unpublished manuscripts investigating the fWHR-aggression relationship. This process identified a collection of 131 articles (as of December 15^th^, 2014).

Next, we reviewed the abstracts of the collected papers and selected articles in which men’s fWHR was examined as a predictor of their personality or behavior (as opposed to how fWHR may affect others’ perceptions of these men); studies examining women’s fWHR were not included. This culling resulted in a sample of 33 articles.

**Fig 1:**
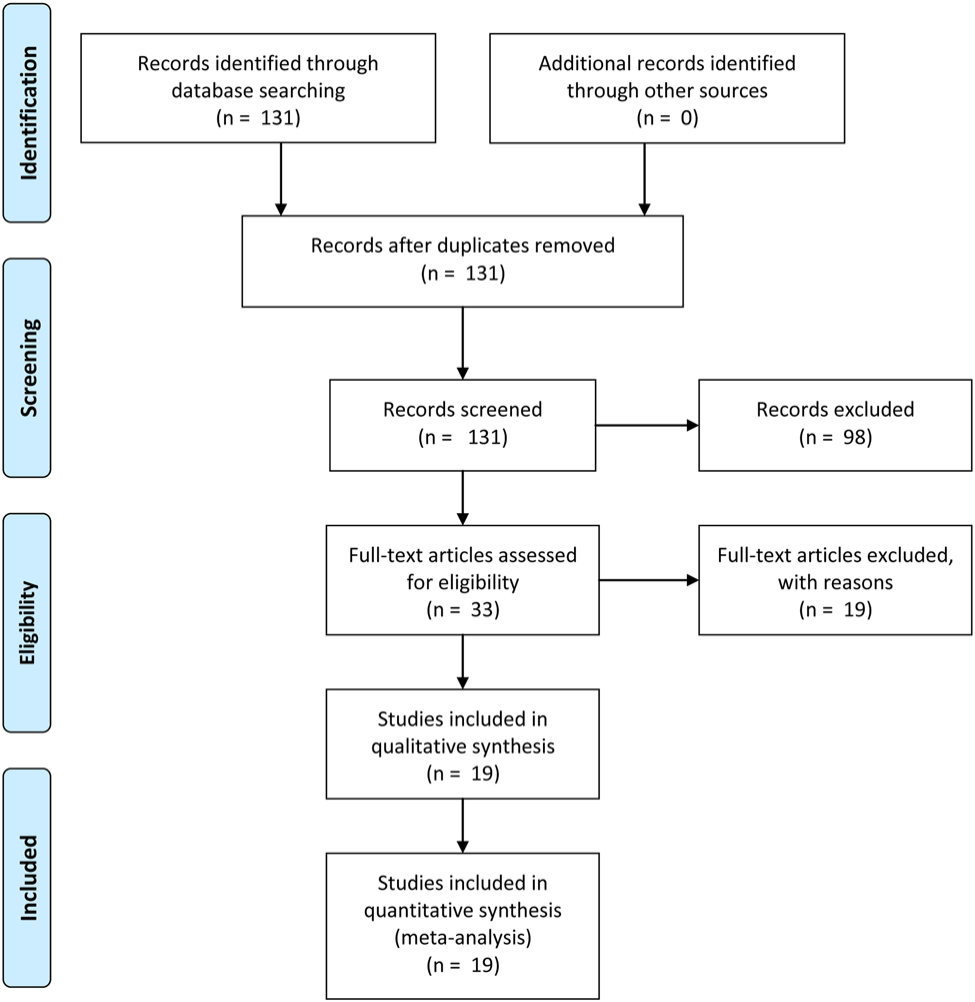
PRISMA flow chart. PRISMA flowchart detailing selection of studies included in meta-analysis.

Finally, we read each paper to determine whether the dependent measure captured aggression. We included both scale measures of aggression, as well as direct behavioral measures (e.g., physical violence). In addition, we included articles in which the authors theorized that the dependent measure represented aggressive behavior (e.g., deception, untrustworthiness). Below, we conduct analyses both including and excluding these additional studies. These selection processes resulted in a final collection of 14 articles, [2, 6–7, 17–26], 19 studies, 32 effect sizes, and a total reported sample of 4327 participants (Figs 1 and S1; Table 1). We adopted correlation coefficients as our common measure of effect sizes.

**Table 1:** Included studies.

Paper	Effect size	Sample size	Sample location	Measure of aggression (Direct vs. Indirect)	Method of facial height measurement	Type of study	Notes
Carré, J.M., & McCormick, C.M. (2008). In your face: Facial metrics predict aggressive behavior in the laboratory and in varsity and professional hockey players. *Proceedings of the Royal Society B*: *Biological Sciences*, *275*, 2651–2656.	0.380	37	Canada	Retaliation in game context (Direct)	Upper lip to mid-brow	Laboratory	
Carré, J.M., & McCormick, C.M. (2008). In your face: Facial metrics predict aggressive behavior in the laboratory and in varsity and professional hockey players. *Proceedings of the Royal Society B*: *Biological Sciences*, *275*, 2651–2656.	0.540	21	Canada	Penalties in hockey games (Direct)	Upper lip to mid-brow	Field	
Carré, J.M., & McCormick, C.M. (2008). In your face: Facial metrics predict aggressive behavior in the laboratory and in varsity and professional hockey players. *Proceedings of the Royal Society B*: *Biological Sciences*, *275*, 2651–2656.	0.300	112	Canada	Penalties in hockey games (Direct)	Upper lip to mid-brow	Field	
Stirrat, M. & Perrett, D.I. (2010). Valid facial cues to cooperation and trust: Male facial width and trustworthiness. *Psychological Science*, *21*, 349–354.	0.400	36	United Kingdom	Untrustworthy actions in economic games (Indirect)	Upper lip to upper eyelid	Laboratory	
Deaner, R.O., Goetz, S.M.M., Shattuck, K. & Schnotala, T. (2012). Body weight, not facial width-to-height ration, predicts aggression in pro hockey players. *Journal of Research in Personality*, *46*, 235–238.	0.062	495.5	Canada; United States	Penalties in hockey games (Direct)	Upper lip to mid-brow	Field	
Haselhuhn, M.P. & Wong, E.M. (2012). Bad to the bone: Facial structure predicts unethical behaviour. *Proceedings of the Royal Society B*: *Biological Sciences*, *279*, 571–576.	0.305	51	United States	Deception in negotiation (Indirect)	Upper lip to mid-brow	Laboratory	Zero-order correlations obtained from author
Haselhuhn, M.P. & Wong, E.M. (2012). Bad to the bone: Facial structure predicts unethical behaviour. *Proceedings of the Royal Society B*: *Biological Sciences*, *279*, 571–576.	0.362	50	United States	Cheating (Indirect)	Upper lip to mid-brow	Laboratory	
Ozener, B. (2012). Facial width-to-height ratio in a Turkish population is not sexually dimorphic and is unrelated to aggressive behavior. *Evolution and Human Behavior*, *33*, 169–173.	-0.006	108	Turkey	Scale measure (Direct)	Upper lip to mid-brow	Laboratory	
Stirrat, M., Stulp, G. & Pollet, T.V. (2012). Male facial width is associated with death by contact violence: Narrow-faced males are more likely to die from contact violence. *Evolution and Human Behavior*, *33*, 551–556.	0.188	523	United States	Death by contact violence (Indirect)	Not Indicated	Field	
Carré, J.M., Murphy, K.R. & Hariri, A.R. (2013). What lies beneath the face of aggression? *Social Cognitive and Affective Neuroscience*, *8*, 224–229.	-0.040	27	United States	Scale measure (Direct)	Upper lip to mid-brow	Laboratory	Zero-order correlations obtained from author
Goetz, S.M.M., Shattuck, K.S., Miller, R.M., Campbell, J.A., Lozoya, E., Weisfeld, G.E. & Carré, J.M. (2013). Social status moderates the relationship between facial structure and aggression. *Psychological Science*, *24*, 2329–2334.	0.080	868	United States	Penalties in hockey games (Direct)	Not Indicated	Field	
Goetz, S.M.M., Shattuck, K.S., Miller, R.M., Campbell, J.A., Lozoya, E., Weisfeld, G.E. & Carré, J.M. (2013). Social status moderates the relationship between facial structure and aggression. *Psychological Science*, *24*, 2329–2334.	0.187	113	United States	Retaliation in game context (Direct)	Not Indicated	Laboratory	
Gomez-Valdes, J., Hunemeir, T., Quinto-Sanchez, M., Paschetta, C., de Azevendo, S., Gonzalez, M.F., Martinez-Abadias, N., Esparza, M., Pucciarelli, H.M., Salzano, F.M., Bau, C.H.D., Bortolini, M.C., Gonzalez-Jose, R. (2013). Lack of support for the association between facial shape and aggression: A reappraisal based on a worldwide population genetics perspective. *PLoS ONE*, *8*: e52317.	0.00	163	Mexico	Severity of crime (Indirect)	Not Indicated	Field	
Geniole, S.N., Keyes, AE., Carré, J.M. & McCormick, C.M. (2014). Fearless dominance mediates the relationship between the facial width-to-height ratio and willingness to cheat. *Personality and Individual Differences*, *54*, 59–64.	0.230	127	Canada; United States	Cheating (Indirect)	Upper lip to mid-brow	Laboratory	
Lefevre, C.E., Etchells, P.J., Howell, E.C., Clark, A.P., & Penton-Voak, I.S. (2014). Facial width-to-height ratio predicts self-reported dominance and aggression in males and females, but a measure of masculinity does not. Biology Letters, 10: 20140729.	0.270	54	United Kingdom	Scale measure (Direct)	Upper lip to upper eyelid	Laboratory	
Třebický, V., Fialová, J., Kleisner, K., Roberts, S. C., Little, A. C., & Havlíček, J. (2014) Further evidence for links between facial width-to-height ratio and fighting success: Commentary on Zilioli et al. (2014). *Aggressive Behavior*. DOI: 10.1002/ab.21559	0.114	146	Varied	Fighting success (Direct)	Upper lip to mid-brow	Field	
Mills, J. (in press). CEO facial width predicts firm financial policies. *Journal of Accounting Research*.	0.033	968	United States	Financial misconduct (Indirect)	Upper lip to mid-brow	Field	
Zilioli, S., Sell, A.N., Stirrat, M., Jagore, J., Vickerman, W. & Watson, N.V. (in press). Face of a fighter: Bizygomatic width as a cue of formidability. *Aggressive Behavior*.	0.154	241	Varied	Fighting success (Direct)	Upper lip to mid-brow	Field	

List of studies included in meta-analysis.

### Data Considerations

A number of studies reported two or more effect sizes. In most cases, we followed common procedure (e.g., [27]) and averaged the two effect and sample sizes. Two papers reported the overall score of an aggression scale and effect sizes for subscales of the larger measure. In these cases, we included only the effect size related to the overall scale.

A few studies had partially overlapping samples. Because the samples were not entirely interdependent, we conducted analyses both including all overlapping studies, as well as including only the study with the largest sample size from each overlapping set [27].

Finally, a paper purporting to demonstrate a null relationship between fWHR and aggression [20] did not present a full report of the study results; an attempt to recover this information from the authors was unsuccessful. To avoid bias in our results from excluding this null finding, we included the study with an effect size of 0.

## Results

To conduct our analysis, we transformed the correlations using Fisher’s Z_r_-transform and weighted the effect size of each study based on sample size. The sample-size weighted average correlation between fWHR and aggression was *r* = .11 (k = 18, N = 4141, 95% CI:.08, .14, Z = 7.06, *p*<.001). This suggests a small [28] but significant correlation between fWHR and aggression. The pattern and significance of these results were unchanged correcting for interdependent samples, *r* = .11, k = 15, N = 3387, 95% CI:.08; .14, Z = 6.43, *p*<.001. Likewise, the results were unchanged when only those studies explicitly capturing aggressive tendencies or behavior were included, *r* = .11, k = 11, N = 2746.5, 95% CI: .07; .15, Z = 5.23, *p*<.001; the results were also significant including only those studies with indirect measures of aggression, *r* = .11, k = 7, N = 1395, 95% CI: .06; .15, Z = 4.75, *p*<.001.

Although the majority of the papers included here reported the measurement of facial height as following the procedures outlined in Carré and McCormick [6] (measuring the distance from the upper lip to the mid-brow), a number of papers either measured facial height as the distance between the upper lip and highest point of the eyelids, or did not specify how they conducted their measurements (see Table 1 for details). In order to determine whether the method of measurement affected our results, we conducted separate analyses for those employing Carré and McCormick’s method versus all others. The average effect size of studies following Carré and McCormick’s method was small and significant (*r* = .10, k = 12, N = 2383.5, 95% CI: .6; .14, Z = 4.84, *p*<.001), as was the average effect size of those studies that followed different procedures or did not specify their procedures, *r* = .12, k = 6, N = 1757, 95% CI: .08; .17, Z = 5.21, *p*<.001. Thus, the method of facial height measurement did not appear to unduly influence the overall results.

In addition, there was variety in the type of study, whereby some studies were conducted in a controlled laboratory setting, whereas others were conducted with field or archival data. In comparing the study settings, we observed a strong average effect size for studies conducted in the laboratory, *r* = .21, k = 9, N = 603, 95% CI: .13; .29, Z = 5.12, *p*<.001. In comparison, the average effect size for field or archival studies was substantially smaller, though still significant, *r* = .09, k = 9, N = 3538, 95% CI: .06; .13, Z = 5.55, *p*<.001.

**Fig 2:**
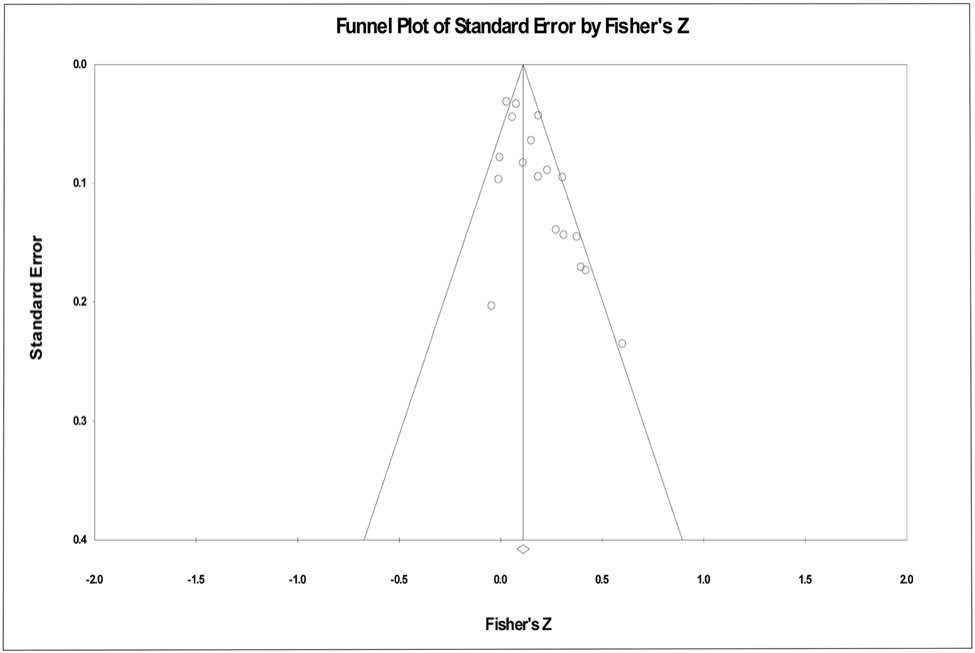
Funnel plot. Funnel plot of Z-transformed effect sizes, plotted against their standard error.

Finally, to address potential concerns of publication bias affecting these results, we constructed a funnel plot of the Z-transformed effect sizes, plotted against their standard error (Fig 2). Although the general shape of the distribution is as expected, with effect sizes with smaller standard errors clustering close to the mean, a visual inspection suggests that a greater number of small or negative effect sizes should have been observed among studies with smaller samples. To detect the risk of potential publication bias, we followed Orwin’s [29] suggested procedure to determine the number of additional studies showing null effects that would need to be included in order for the mean effect size to reduce substantially (in this case, in half). The results of this analysis suggest that an additional 21.6 studies with null results would need to be included, indicating that publication bias toward large, significant effects is unlikely to fully drive the observed results. Taken together, these analyses suggest a small, but robust, positive association between fWHR and aggression.

## Discussion

Despite mixed findings in the literature, results of our meta-analysis demonstrate a robust positive link between fWHR and aggression suggesting that fWHR is a reliable marker (and signal) of aggression in men. This analysis ameliorates some of the concerns raised regarding extant research, such as the use of small samples to examine this relationship in some previous studies.

Of course, meta-analytic procedures involve tradeoffs. One concern with this method is the “file drawer effect” by which studies reporting significant results are more likely to be published relative to those reporting null results. Indeed, our funnel plot suggests that null or negative correlations between fWHR and aggression may be underrepresented in studies with smaller sample sizes. We attempted to account for the file drawer problem in our study in a number of ways. For instance, we requested unpublished manuscripts from a number of scholars in the field in order to obtain studies that had not appeared in journals; we were not able to obtain access to any such studies. In addition, we calculated the number of null studies that would need to be included in our sample in order for the observed effect to be halved. The large number of studies required suggests that the relationship between fWHR and aggression found in our meta-analysis is not unduly influenced by the absence of unpublished null findings.

Another cost-benefit tradeoff of a meta-analysis is the ability to compile studies with varied methods and samples. On the one hand, analyzing studies at the effect size level allows for a broad synthesis of the literature that would otherwise be impossible. On the other hand, such an analysis masks substantial differences across various studies that could have significant implications for the area of research. For example, researchers have employed a wide array of dependent measures in their investigation of the fWHR-aggression relationship, some directly measuring aggression, others ostensibly indirectly tapping into the construct. Similarly, differences in the measurement of fWHR (specifically, the facial height component) may make direct comparisons problematic. In our analysis, we attempted to address potential concerns by conducting supplementary analyses to examine the potential impact of these differences. Although we found consistent support of a positive fWHR-aggression link, future reviews should continue to account for these factors as the literature develops.

We have primarily highlighted the robustness of the relationship between fWHR and aggression, but it is important to note that the effect size is small. The implication, of course, is that a substantial percentage of the variance in explaining men’s aggressive tendencies is explained by other factors. This is a critical point, as researchers have demonstrated that observers rely on men’s facial structure as an honest signal of character and behavioral intentions (e.g., [2, 12–13]). As described above, the average effect size was substantially higher for studies conducted in a controlled, laboratory setting compared to those conducted in the field. The weaker relationship in more natural settings emphasizes the caution that should be taken in making strong predictions of a man’s behavior based on his face.

Finally, these findings beg the question of why previous support for this link is inconsistent. Some researchers have conducted studies of the boundary conditions of the fWHR-aggression relationship with intriguing results. For example, the relationship between fWHR and aggression appears to be particularly strong among men of relatively low social status [24]. Further work on other moderating factors, such as cultural differences, should be equally fruitful. More broadly, examining social contextual factors that might influence this fWHR-aggression link is warranted.

## Supporting Information

S1 Fig PRISMA checklist. (DOC)
